# An integrative computational and experimental evaluation of natural compounds with potential to target LuxS in *Klebsiella pneumoniae*

**DOI:** 10.1007/s00203-026-05191-z

**Published:** 2026-09-15

**Authors:** Mashego Jackson Mamosebo, Suheena Ayrga, Shabir A. Madhi, Shama Khan

**Affiliations:** 1 Department of Clinical Microbiology and Infectious Diseases, Faculty of Health Sciences, University of the Witwatersrand, Johannesburg, South Africa; 2 South African Medical Research Council, Vaccines and Infectious Diseases Analytics Research Unit, Faculty of Health Sciences, University of the Witwatersrand, Johannesburg, South Africa; 3 Wits Infectious Diseases and Oncology Research Institute, Faculty of Health Sciences, University of the Witwatersrand, Johannesburg, 2050 South Africa; 4 Unit for Environmental Sciences and Management, North-West University, Potchefstroom Campus, Potchefstroom, 2520 South Africa

**Keywords:** *Klebsiella pneumoniae*, Autoinducer-2 (AI-2), Quorum sensing, Biofilms, Antimicrobial resistance

## Abstract

*Klebsiella pneumoniae* (*KPn*) has emerged as a major global health concern due to increasing antimicrobial resistance (AMR). The development of new antimicrobial agents is essential to address multidrug-resistant and emerging hypervirulent strains associated with increased morbidity and mortality worldwide. In this study, an integrative computational–experimental approach was employed to characterise the anti-quorum sensing and anti-biofilm activities of selected natural compounds targeting the LuxS/autoinducer-2 (AI-2) system in *KPn*. Six compounds were initially screened using homology modelling, molecular docking, pharmacophore analysis, and molecular dynamics (MD) simulations. Resveratrol, quercetin, and epigallocatechin gallate showed favourable binding to LuxS. Although quercetin (ΔG_bind = − 45.47 kcal/mol) and epigallocatechin gallate (− 43.04 kcal/mol) exhibited stronger predicted affinities than resveratrol (− 39.22 kcal/mol), resveratrol demonstrated superior complex stability (RMSD: 1.8 ± 0.2 Å) and persistent interactions with key residues (Tyr63, Trp68). Experimental validation demonstrated that resveratrol exhibited significant antimicrobial activity (MIC/MBC = 32 µg/mL), significantly inhibited quorum sensing-associated phenotypes, and reduced *KPn*-*Acinetobacter baumannii* (*ABa*) dual-species biofilm formation inhibition by 54.7%. These findings identify resveratrol as a promising anti-virulence candidate and highlight the utility of integrative computational and experimental strategies for the identification of compounds targeting quorum sensing and biofilm formation inhibition in multidrug-resistant pathogens.

**Graphical abstract:**

**Figure:**
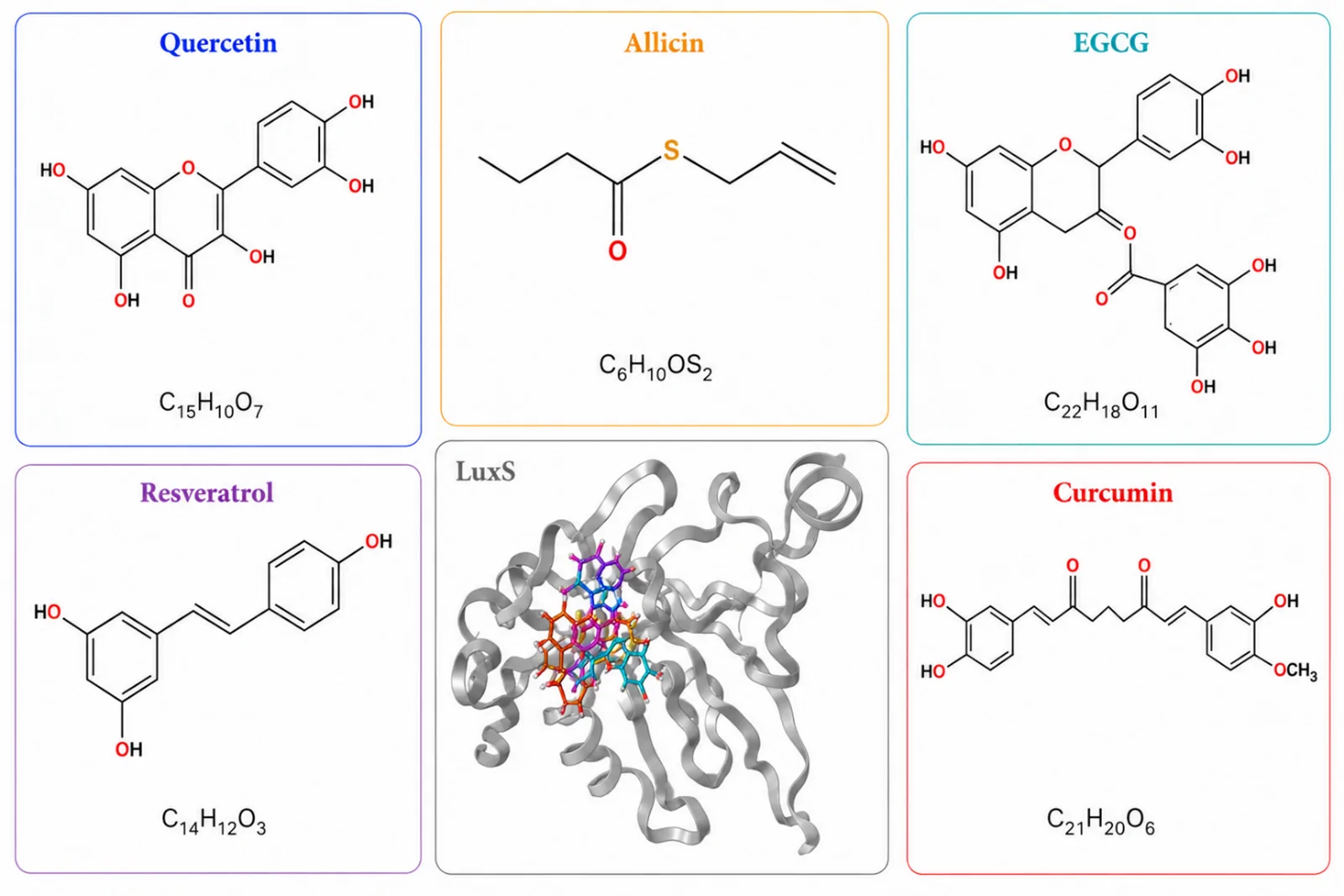
Graphical abstract showing natural compounds investigated in this study and their interaction with the LuxS protein, a key regulator of quorum sensing in *Klebsiella pneumoniae*

## Introduction

Antimicrobial resistance (AMR) in bacterial infections is estimated to be associated with approximately 5.0 million deaths annually, of which 4.3 million occur in low- and middle-income countries (LMICs) (Murray et al. 2022). The escalating crisis of morbidity and mortality due to AMR bacteria has rendered many conventional antibiotics increasingly ineffective, necessitating the development of alternative therapeutic strategies that extend beyond traditional bactericidal approaches (Bengtsson-Palme et al. 2018).

The ESKAPE pathogens (*Enterococcus faecium*,* Staphylococcus aureus*,* Klebsiella pneumoniae*,* Acinetobacter baumannii*,* Pseudomonas aeruginosa*, and *Enterobacter species*) are among the most clinically significant AMR bacterial pathogens. In particular, *Klebsiella pneumoniae* (*KPn*) has been designated a critical priority pathogen by the World Health Organization (WHO) (World Health Organization 2024). It is a Gram-negative bacterium, is a major cause of hospital-acquired infections, including pneumonia, bloodstream infections, urinary tract infections, and meningitis, with case fatality risk exceeding 40% when associated with multidrug-resistant (MDR) strains (Chen et al. 2024). The prominence of *KPn* is attributed not only to extensive antimicrobial resistance mechanisms, including extended-spectrum β-lactamases and carbapenemases (Liang et al. 2022), but also to its ability to form biofilms that enhance persistence, immune evasion, and horizontal gene transfer (Olwagen et al. 2025).

Biofilm formation inhibition and virulence in *KPn* are closely associated with quorum sensing (QS), a cell-density-dependent communication system that regulates gene expression (Deryabin et al. 2019; Khan et al. 2023). Unlike many Gram-negative bacteria that rely on acyl-homoserine lactone (AHL)-based systems, *KPn* predominantly utilises the LuxS/autoinducer-2 (AI-2) pathway for intercellular communication (Chen et al. 2020). The LuxS enzyme catalyses the production of AI-2, linking quorum sensing with central metabolism through the activated methyl cycle (Schauder et al. 2001). The AI-2 system plays a key role in regulating biofilm formation, virulence factor expression, and plasmid-mediated gene transfer thereby making LuxS an attractive target for anti-virulence therapy (Hall-Stoodley et al. 2004).

Multiple strategies have been investigated to disrupt the LuxS/AI-2 system at distinct mechanistic levels, including substrate analogues such as S-adenosyl-L-homocysteine (SAH), which competitively inhibits LuxS by mimicking its natural substrate S-ribosylhomocysteine (SRH), thereby reducing AI-2 synthesis. Additional approaches involve synthetic DPD analogues that interfere with AI-2 receptor binding, ribose-based intermediates that disrupt catalytic function through structural mimicry, and metal-chelating agents that impair the zinc-dependent enzymatic activity of LuxS (Roy et al. 2011). Collectively, these strategies reinforce the feasibility of targeting LuxS as a means of modulating quorum sensing and attenuating virulence without directly affecting bacterial viability.

Targeting QS represents a promising strategy to disarm *KPn* without exerting strong selective pressure for resistance (Hetta et al. 2024; Silva-Bea et al. 2026). Natural compounds, including flavonoids and polyphenols such as quercetin, resveratrol, epigallocatechin gallate (EGCG), and curcumin, have demonstrated the ability to interfere with QS signaling, disrupt biofilms, and attenuate virulence through diverse mechanisms (Rutherford and Bassler 2012). Nevertheless, experimental screening of large compound libraries is resource-intensive, highlighting the importance of computational approaches for prioritising promising candidates.

Although several natural compounds, including resveratrol, quercetin and EGCG, have previously been reported to exhibit anti-quorum sensing and antibiofilm activities, their effects have largely been evaluated through phenotypic assays without systematically investigating their interactions with the LuxS target in *KPn*. Furthermore, studies integrating structure-based computational screening with comprehensive experimental validation remain limited. Therefore, there is a need for an integrated workflow that combines molecular modelling, pharmacophore analysis, molecular dynamics simulations, ADMET prediction and biological validation to efficiently prioritise and characterise potential LuxS-targeting anti-virulence agents.

In this study, an integrative computational-experimental approach was employed to identify and evaluate natural compounds with the potential to target the LuxS enzyme in *KPn*. Structure-based computational methods, including homology modelling, molecular docking, pharmacophore modelling, MD simulations and ADMET prediction were used to prioritise candidate compounds for experimental evaluation. These compounds were subsequently assessed through in vitro antimicrobial, quorum-sensing interference and anti-biofilm assays. Quorum-sensing interference was evaluated using a *Chromobacterium violaceum* (*C. violaceum*) biosensor system, in which inhibition of violacein production served as a phenotypic indicator of QS disruption. While this system primarily reflects AHL-mediated signalling, it was used as a general phenotypic screening tool for QS interference rather than as direct evidence of LuxS/AI-2 pathway inhibition in *KPn*. S-adenosyl-L-homocysteine (SAH) was employed as a reference ligand because of its reported association with the LuxS/AI-2 pathway, enabling comparison of the candidate compound with a metabolically relevant reference molecule (Wang et al. 2019). By integrating target-based computational prioritisation with phenotypic validation, this study provides a rational workflow for identifying potential anti-virulence agents targeting quorum sensing and biofilm formation inhibition in MDR *KPn*.

## Methods and materials

This study employed an integrative computational-experimental approach to characterise natural compounds with anti-quorum-sensing (QS) and anti-biofilm activity against *KPn*. The workflow comprised (i) in silico modelling and screening targeting the LuxS enzyme, followed by (ii) in vitro validation of selected compounds using antimicrobial, anti-QS, and biofilm formation inhibition assays. This combined strategy enabled both mechanistic prediction and functional validation of anti-virulence-associated activity.

### In silico predictions

#### Software and computational resources

Molecular docking and molecular modelling were performed using the Schrödinger Maestro suite (Release 2023-2, Schrödinger LLC, USA). Molecular dynamics (MD) simulations were conducted using the AMBER 18 package with GPU acceleration. All computations were performed on the Centre for High-Performance Computing (CHPC), Cape Town, South Africa.

#### Target sequence retrieval and template selection

The amino acid sequence of S-ribosylhomocysteine lyase (LuxS) from *KPn* (UniProt accession: B5XVC4) was retrieved from UniProtKB. Protein databank entry 5E68, a 1.58 Å crystal structure of LuxS from *Salmonella enterica* serovar Typhi, was selected as the template for homology modelling based on its high sequence identity (91%), coverage (98%), and significant E-value (2 × 10⁻⁸⁰).

#### Homology modelling and validation

Homology modelling was conducted using the Prime module in Schrödinger. Sequence alignment was performed using ClustalW, and model construction employed an energy-based approach incorporating loop refinement and side-chain optimisation. The zinc cofactor present in the template was retained in the model. Model validation was performed using the SAVES server (https://saves.mbi.ucla.edu/), including PROCHECK (Ramachandran analysis), ERRAT, VERIFY_3D and WHATCHECK. Structural agreement was further assessed by comparison with the AlphaFold-predicted LuxS structure. These analyses were used to assess the overall structural quality of the model prior to downstream computational analysis.

#### Protein and ligand preparation

The LuxS model was prepared using the Protein Preparation Wizard in Schrödinger. Hydrogen atoms were added, bond orders assigned, and protonation states optimized at physiological pH using Epik (Shelley et al. 2007). Energy minimisation was conducted using the OPLS-2005 (41) force field with a convergence threshold of 0.30 Å RMSD. Six natural compounds (quercetin, curcumin, epigallocatechin gallate (EGCG), allicin, resveratrol, and berberine) were selected based on reported QS inhibitory activity, structural diversity, availability and representation of different classes of natural products, including flavonoids, polyphenols, organosulfur compounds, and alkaloids. Rather than performing a broad high-throughput virtual screening, this focused selection enabled detailed computational characterisation and subsequent experimental validation of representative compounds with established anti-virulence potential. S-adenosyl-L-homocysteine (SAH) was included as a reference LuxS ligand. Ligands were prepared using LigPrep, including ionisation state prediction (pH 7.0 ± 2.0), tautomer generation, stereoisomer enumeration, desalting, and energy minimisation using the OPLS-2005 force field.

#### Identification of pharmacophore hypothesis

A structure-based e-pharmacophore modelling approach was employed using the PHASE module (42). Six standard pharmacophoric features were considered: hydrogen bond acceptors (A), hydrogen bond donors (D), hydrophobic groups (H), negatively ionisable groups (N), positively ionisable groups (P), and aromatic rings (R). An energy-optimised pharmacophore (e-pharmacophore) was derived based on the docked conformation of S-adenosyl-L-homocysteine (SAH) within the LuxS active site. The model incorporated key interaction features, including hydrogen bonding and hydrophobic interactions. Excluded volumes were defined around the bound ligand to represent steric constraints of the active site. Pharmacophore features were selected based on critical interactions with key active-site residues involved in ligand binding. The resulting pharmacophore model captured the essential structural features required for effective interaction with the LuxS active site (Dixon et al. 2006).

#### Molecular docking

The Glide-based docking module of the Schrödinger suite was employed to identify compounds with strong binding affinity toward the LuxS protein. A receptor grid was generated around the active site using an outer grid box of 30 × 30 × 30 Å and an inner grid box of 18 × 18 × 18 Å to ensure accurate ligand sampling within the binding pocket. The compound library was subjected to a hierarchical two-stage docking protocol, comprising Standard Precision (SP) followed by Extra Precision (XP) docking. Top candidates were selected based on XP GlideScore ranking and favourable interaction profiles. A cutoff of -6 kcal/mol was applied, and compounds with GlideScores ≤ -6 kcal/mol were retained for further analysis. Complexes exhibiting more negative binding scores and consistent interactions with key active-site residues were prioritised, indicating higher predicted binding stability. Berberine did not localise within the catalytic pocket and consistently occupied alternative regions, suggesting a different binding preference, and was therefore excluded from downstream analysis. Because the LuxS structure used in this study was generated by homology modelling rather than obtained as an experimentally determined co-crystal structure containing a native ligand, conventional docking validation through ligand redocking and RMSD analysis could not be performed. Instead, the reliability of the computational workflow was supported by rigorous validation of the homology model using PROCHECK, ERRAT, VERIFY3D and ProSA, together with structural comparison against the AlphaFold-predicted LuxS structure. Furthermore, the stability of the predicted protein–ligand complexes were evaluated through 100 ns molecular dynamics simulations. These simulations provided additional assessment of complex stability but were not considered a substitute for conventional docking validation. Future studies using experimentally resolved LuxS–ligand structures will enable conventional redocking and RMSD-based validation.

#### ADMET and drug-likeness prediction

Pharmacokinetic profiles were predicted using QikProp 5.6 including molecular weight, hydrogen bond donors and acceptors, octanol/water partition coefficient (logP), polar surface area (PSA), aqueous solubility (logS), Caco-2 cell permeability, blood–brain barrier penetration (logBB), human oral absorption and potential cardiotoxicity (hERG channel inhibition). Drug-likeness was assessed using Lipinski’s Rule of Five (Lipinski et al. 2001).

#### Molecular dynamics (MD) simulations

All atom MD simulations(Salsbury 2010) were performed for selected compounds (resveratrol, quercetin, EGCG) and SAH using AMBER18 tools (Case et al. 2023). Systems were parameterised using the ff14SB force field for the protein(Maier et al. 2015) and General AMBER Force Field (GAFF) for ligands, solvated in a TIP3P water box, and neutralised with counterions. Long-range electrostatic interactions were treated using the Particle Mesh Ewald (PME) method with a non-bonded cut-off of 12 Å. The SHAKE algorithm constrained all bonds involving hydrogen atoms, enabling a 2-fs integration time step. Energy minimisation was performed in two stages: 2500 steps of steepest descent followed by 1500 steps of conjugate gradient minimisation, initially with positional restraints that were gradually reduced. Systems were heated from 0 K to 300 K under constant volume with weak harmonic restraints (10 kcal/mol/Å²) followed by density equilibration (50 ps, Berendsen barostat) and constant-pressure equilibration (500 ps, 1 atm, 300 K). Each protein–ligand complex was subjected to a 100 ns MDS under NPT conditions. The resulting trajectories were analysed using RMSD, RMSF, radius of gyration, solvent-accessible surface area, hydrogen-bond interactions, and other structural parameters to assess complex stability. As a single trajectory was generated for each complex, the MD results were interpreted as comparative, predictive evidence of complex stability rather than definitive quantitative measurements. Independent replicate simulations and formal convergence analyses were not performed. Trajectories were analysed using CPPTRAJ for RMSD, RMSF, radius of gyration, and solvent-accessible surface area (Roe and Cheatham 2013).

#### Binding free energy calculations (MM/GBSA)

Binding free energy was estimated using the MM/GBSA method from the final 50 ns of MD trajectories (Genheden and Ryde 2015). The MM/GBSA calculations were used to comparatively estimate the relative binding energetics of the protein–ligand complexes. Given that a single MD trajectory was generated for each complex and formal convergence or block-averaging analyses were not performed, the calculated binding free energies were interpreted as comparative estimates. The binding free energy ($\:{\Delta\:}{G}_{\mathrm{bind}}$) was calculated as:

$$\:{\Delta\:}{G}_{\mathrm{bind}}={G}_{\mathrm{complex}}-{G}_{\mathrm{protein}}-{G}_{\mathrm{ligand}}$$

where $\:G={E}_{\mathrm{gas}}+{G}_{\mathrm{solvation}}-TS$. The entropic term (* -TS*) was excluded as it is computationally expensive and contributes minimally to relative binding energy comparisons. The gas-phase energy ($\:{E}_{\mathrm{gas}}$) comprises internal, van der Waals, and electrostatic contributions:

$$\:{E}_{\mathrm{gas}}={E}_{\mathrm{int}}+{E}_{\mathrm{vdW}}+{E}_{\mathrm{elec}}$$

Solvation free energy ($\:{G}_{\mathrm{solvation}}$) includes polar and non-polar components:

$$\:{G}_{\mathrm{solvation}}={G}_{\mathrm{polar}}+{G}_{\mathrm{nonpolar}}$$

Polar solvation energy was calculated using the Generalized Born (GB) model (dielectric constants: 1.0 for solute, 80.0 for solvent). Non-polar solvation energy was estimated from SASA:

$$\:{G}_{\mathrm{nonpolar}}=\gamma\:\cdot\:\mathrm{SASA}+\beta\:$$

where $\:\gamma\:=0.00542$ kcal·$\:{\mathrm{m}\mathrm{o}\mathrm{l}}^{-1}$·$\:{\mathrm{\AA\:}}^{-2}$ and $\:\beta\:=0$ kcal·$\:{\mathrm{m}\mathrm{o}\mathrm{l}}^{-1}\left(54\right)$.

### In vitro experiments

#### Bacterial strains, culture conditions and compound preparations

Compounds selected from computational screening were prioritised for experimental validation based on binding affinity, stability, and predicted pharmacokinetic properties. A reference strain (*KPn* ATCC 13883), a clinical multidrug-resistant (MDR) *KPn* isolate and a clinical MDR *Acinetobacter baumannii* (*ABa*) isolate were used to assess biofilm formation inhibition (Thakur et al. 2025). The clinical isolates were obtained through a pathogen surveillance study conducted at the Wits VIDA research unit. No patient-identifying information was accessed, and detailed clinical metadata were not available or within the scope of the present study. A biosensor strain, *Chromobacterium violaceum (C. violaceum)*, was used for anti-quorum sensing (AQS) assays. Cultures were maintained as glycerol stocks at − 80 °C and revived on LB or BHI agar. Working cultures were prepared in Mueller-Hinton broth (MHB) or Luria-Bertani (LB) broth and standardised to OD₆₀₀ = 0.1 (~ 10⁸ CFU/mL). Quercetin, resveratrol, and EGCG were selected based on computational screening. Stock solutions were prepared in DMSO (or PBS for SAH), filter-sterilised, and used at final solvent concentrations ≤ 2% (v/v).

#### Antibacterial activity (MIC and MBC)

Minimum inhibitory concentrations (MICs) were determined using the broth microdilution method in 96-well plates according to CLSI guidelines. Serial dilutions (1024–0.5 µg/mL) were prepared, and bacterial inocula (~ 5 × 10⁵ CFU/mL) were added. After incubation (16–20 h, 35 °C), viability was assessed using p-iodonitrotetrazolium violet (INT). MIC was defined as the lowest concentration preventing colour change. Minimum bactericidal concentrations (MBCs) were determined by plating aliquots from MIC wells onto agar. A ≥ 3 log₁₀ reduction in CFU was considered bactericidal.

#### Anti-quorum sensing assays

Quorum-sensing inhibition was evaluated using the *C. violaceum* biosensor assay, where reduced violacein production serves as a phenotypic indicator of quorum-sensing interference. Since this biosensor predominantly reflects AHL-mediated signalling, the assay was used solely as a general phenotypic screen for quorum-sensing inhibition and should not be interpreted as direct evidence of LuxS/AI-2 pathway inhibition in *KPn*. Because the assay was performed at MIC concentrations, the observed reduction in violacein production may have been influenced by growth inhibition. Therefore, the assay results were interpreted as quorum-sensing-associated phenotypic interference rather than definitive, growth-independent QS inhibition.

#### Qualitative and quantitative violacein assay

Agar well diffusion assays were performed using *C. violaceum*. Wells were loaded with compounds at MIC concentrations, and plates were incubated at 30 °C for 24 h. Zones of pigment inhibition were measured. Violacein inhibition was quantified in microtiter plates. After incubation, pigment was extracted with DMSO, and absorbance was measured at 485 nm. Percentage inhibition was calculated relative to untreated controls, and IC₅₀ values were determined using nonlinear regression.

#### Biofilm formation inhibition assay

Biofilm formation inhibition was assessed in sterile 96-well plates using single- and dual-species models comprising *KPn* ATCC 13,883, a clinical MDR *KPn* isolate, and a clinical MDR *ABa* isolate. Overnight cultures were standardised to OD₆₀₀ = 0.1 (approximately 10⁸ CFU/mL) and subsequently diluted 1:100 in fresh MHB to obtain working inocula of approximately 10⁶ CFU/mL. For dual-species assays, equal volumes of the *KPn* and *ABa* working inocula were mixed immediately before inoculation. Compounds were tested at sub-MIC concentrations during the biofilm formation period and plates were incubated statically at 37 °C for 24 h. Biofilm biomass was quantified by crystal violet staining, followed by absorbance measurement at 595 nm. Biofilm formation inhibition (%) was calculated relative to untreated controls, and the minimum biofilm inhibitory concentration (MBIC) was defined as the lowest concentration producing ≥ 50% inhibition of biofilm formation. This assay evaluated inhibition of biofilm formation and did not assess eradication of pre-established mature biofilms.

#### Viable cell counting on selective media

Following 24 h biofilm formation, selected wells were collected before crystal violet staining. Biofilms were resuspended in sterile PBS by vigorous pipetting and scraping, serially diluted ten-fold, and plated onto MacConkey agar for enumeration of *KPn* and CHROMagar Acinetobacter for selective recovery of *ABa*. Plates were incubated at 37 °C for 24–48 h, after which colonies were counted and viable counts were expressed as CFU/mL. Log₁₀ reductions were calculated relative to untreated growth controls.

#### Statistical analysis

All experiments were conducted using three independent biological replicates (*n* = 3). Data are presented as mean ± standard deviation. Statistical analyses were performed using GraphPad Prism 9.0. One-way ANOVA with Dunnett’s test and two-way ANOVA with Tukey’s test were used as appropriate. Pearson correlation analysis was performed to enable integration of computational predictions with experimental outcomes. Statistical significance was set at *p* < 0.05.

## Results and discussion

### In silico results

#### Homology modelling and validation

To our knowledge, the three-dimensional structure of the LuxS protein from *KPn* has not been reported in publicly available databases. Therefore, a homology modelling approach was employed to generate the LuxS structure. The LuxS protein from *Salmonella enterica* (PDB ID: 5E68) was selected as the template due to its high sequence identity (91%) and structural conservation. The generated model exhibited the characteristic TIM-barrel (β/α)₈ fold with the catalytic zinc ion correctly positioned within the active site. Validation of the model showed favourable overall structural quality, with an ERRAT score of 94.27% and 86.42% of residues passing VERIFY3D thresholds. The ProSA Z-score (− 5.62) was within the range for experimentally determined structures, while Ramachandran analysis showed that 91.4% of residues were in the most favoured regions, with no residues in disallowed regions. These results support the overall structural quality of the generated LuxS model for subsequent computational analysis.

#### Molecular docking of natural compounds

Molecular docking was performed to evaluate the binding affinity of selected natural compounds towards the LuxS protein, using S-adenosyl-L-homocysteine (SAH) as a reference ligand (Table 1).

**Table 1:** Glide XP docking scores (kcal/mol) of selected test compounds and the reference ligand SAH against the LuxS active site

Compounds	XP Score (kcal/mol)
Resveratrol	-8.51
Quercetin	-8.01
Epigallocatechin gallate (EGCG)	-7.58
S-adenosyl-L-homocysteine (SAH)	-6.78
Curcumin	-5.21
Allicin	-5.03

Among the tested compounds, resveratrol showed the strongest binding affinity with a docking score of − 8.51 kcal/mol, followed by quercetin (-8.01 kcal/mol) and epigallocatechin gallate (EGCG) (-7.58 kcal/mol). All three compounds exhibited more favourable binding scores compared to SAH (-6.78 kcal/mol), indicating stronger interactions within the active site.

**Fig. 1:**
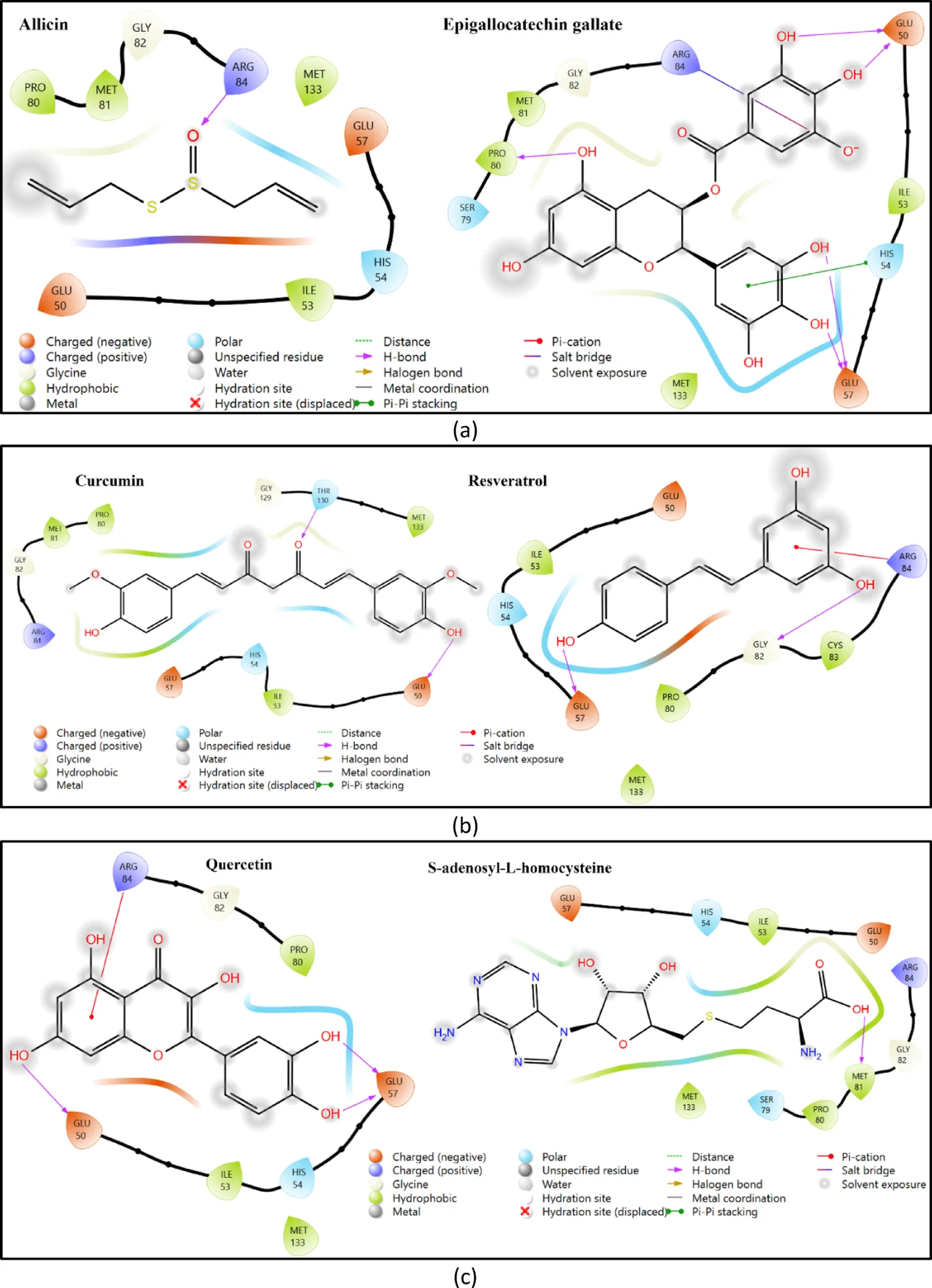
**A**: 2D interaction of Allicin and Epigallocatechin gallate with LuxS active site.** B**: 2D interaction of Curcumin and Resveratrol with LuxS active site. **C**: 2D interaction of Quercetin and S-adenosyl-L-homocysteine with LuxS active site

Two-dimensional interaction maps (Fig. 1A, B, C) illustrate the binding interactions of the selected compounds with key active-site residues of LuxS. These interactions include hydrogen bonds, hydrophobic contacts, and electrostatic interactions involving residues such as Glu50, Glu57, His54, Ile53, Met81, Met133, Pro80, Gly82, and Arg84. Conserved interaction patterns were observed across compounds, with resveratrol showing favourable positioning within the catalytic pocket and consistent hydrogen bonding with key residues. Based on these findings, resveratrol, quercetin, and EGCG were selected for further analysis.

#### Structure-based pharmacophore modelling

**Fig. 2:**
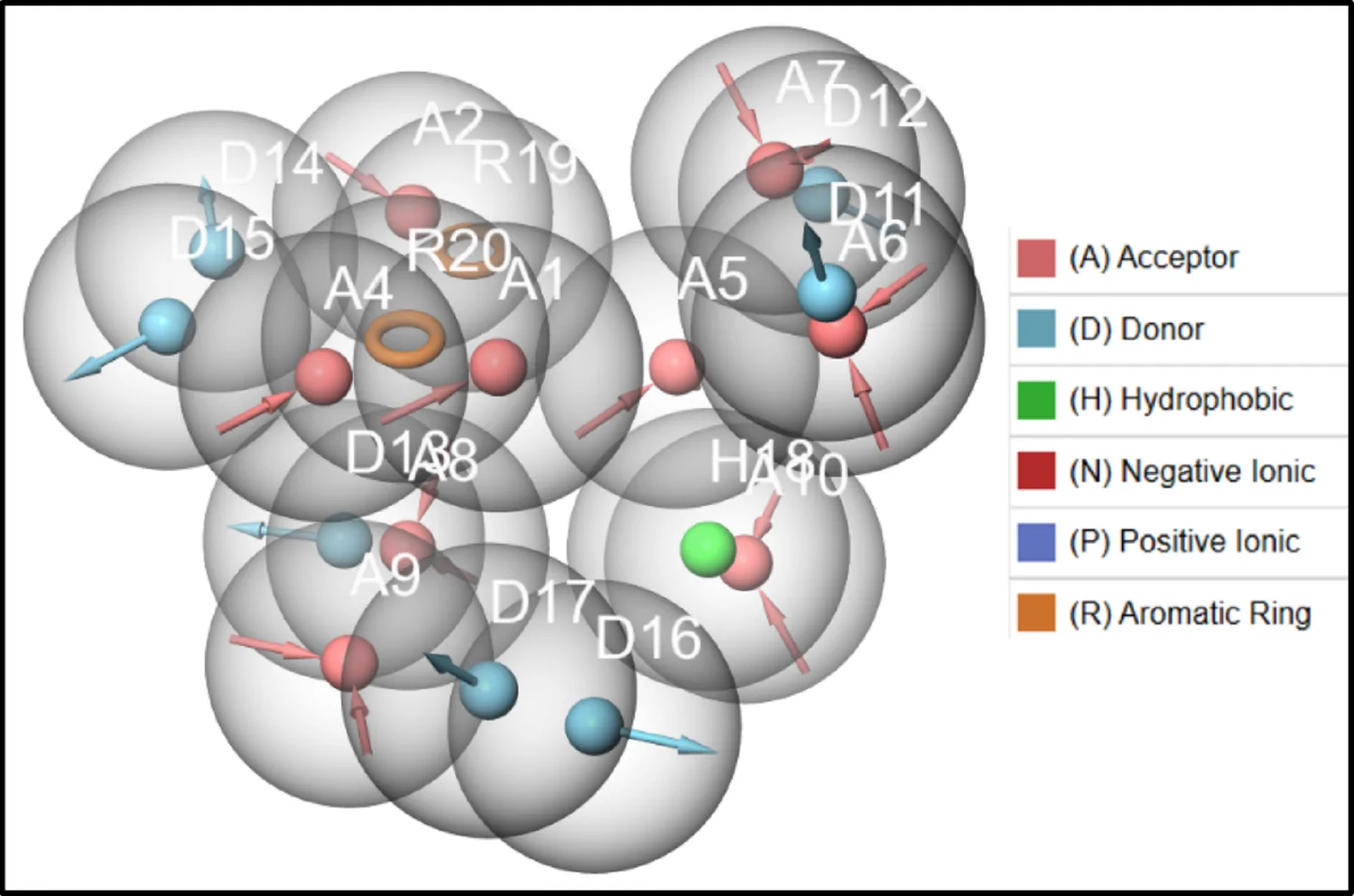
Structure-based e-pharmacophore model of the LuxS active site derived from the bound conformation of SAH. The 3D pharmacophore illustrates key interaction features, including hydrogen bond acceptors (red arrows), hydrogen bond donors (blue arrows), and aromatic ring interactions (orange spheres), with the ligand structure embedded within defined excluded volumes (grey spheres) representing steric constraints

A structure-based e-pharmacophore model was generated using the docked conformation of SAH within the LuxS active site (Fig. 2). The model identified key interaction features, including hydrogen bond donors (blue arrows), hydrogen bond acceptors (red arrows), and aromatic ring interactions (orange spheres). Excluded volumes (grey spheres) represent steric constraints of the binding pocket. The selected compounds, particularly resveratrol and quercetin, showed good alignment with the pharmacophore features, suggesting that these molecules possess the required structural characteristics for effective interaction with the LuxS active site.

#### ADMET analysis

The drug-likeness and pharmacokinetic properties of the selected compounds were evaluated using QikProp. Resveratrol and quercetin complied with Lipinski’s Rule of Five with no violations, indicating favourable oral bioavailability, whereas EGCG and SAH each showed violations, mainly due to higher polarity and molecular size. As shown in Table 2, the qualitative human oral absorption score (scale 1–3) aligned with polar surface area (PSA). Resveratrol (PSA 56.6 Å²) and quercetin (91.9 Å²) were both assigned the highest absorption category (3), while EGCG (PSA 138.1 Å²) was predicted as moderate (2) and SAH (PSA 123.0 Å²) as low (1). Consistent with these trends, Caco-2 permeability (QPPCaco) was exceptionally high for resveratrol (395.7 nm s⁻¹), confirming a strong ability to traverse intestinal epithelia, whereas EGCG (13.7 nm s⁻¹) and SAH (6.3 nm s⁻¹) showed limited permeability. Predicted aqueous solubility (QPlogS) was highest for resveratrol (− 1.15) and quercetin (− 2.50), while EGCG (− 5.80) and SAH (− 2.02) were less soluble. Octanol/water partitioning (QPlogPo/w) reflected the polarity differences, with EGCG being the most lipophilic (2.72) and SAH the most hydrophilic (− 2.49). Brain penetration estimates (CNS activity and QPlogBB) indicated that all compounds are unlikely to enter the CNS; resveratrol gave a modest CNS score of − 1 and a QPlogBB of − 0.81, whereas the others were strongly restricted. The hERG inhibition risk (QPlogHERG) was within acceptable limits. The predicted pharmacokinetic properties were broadly consistent with the experimental findings. Resveratrol exhibited the most favourable ADMET profile, characterised by high predicted intestinal permeability, good oral absorption, and favourable aqueous solubility, which may have facilitated its superior antimicrobial, quorum-sensing interference, and antibiofilm activities observed in vitro. In contrast, although EGCG demonstrated favourable docking and binding free energy, its comparatively higher polarity, larger polar surface area, and lower predicted membrane permeability may have limited its cellular uptake and consequently reduced its biological activity. Quercetin displayed intermediate pharmacokinetic characteristics, consistent with its moderate computational and experimental performance. These observations highlight that predicted binding affinity alone is insufficient to predict biological efficacy and underscore the importance of integrating pharmacokinetic properties with molecular modelling and experimental validation when prioritising candidate anti-virulence compounds.

**Table 2:** In silico ADMET predictions of the selected compounds

Compounds Names	CNS	QPlogKhsa	SASA	QPlogPo/w	QPlogS	QPlogBB	QPlogHERG	QPPCaco	PSA	Rule Of Five	Human Oral Absorption
EGCG	-2	0.616	634.058	2.720	-5.797	-3.345	-5.649	13.723	138.055	2	2
Quercetin	-2	-0.459	455.789	0.409	-2.498	-2.074	-4.796	58.843	91.896	0	3
Resveratrol	-1	-0.615	353.509	0.302	-1.147	-0.807	-3.004	395.735	56.629	0	3
SAH	-2	-1.167	617.131	-2.490	-2.015	-2.461	-6.481	6.310	122.974	2	1

#### Molecular dynamics simulation analysis: structural stability, flexibility, compactness, and solvent exposure

To evaluate the dynamic stability and conformational behaviour of the LuxS protein in complex with selected ligands, 100 ns molecular dynamics (MD) simulations were performed.

**Fig. 3:**
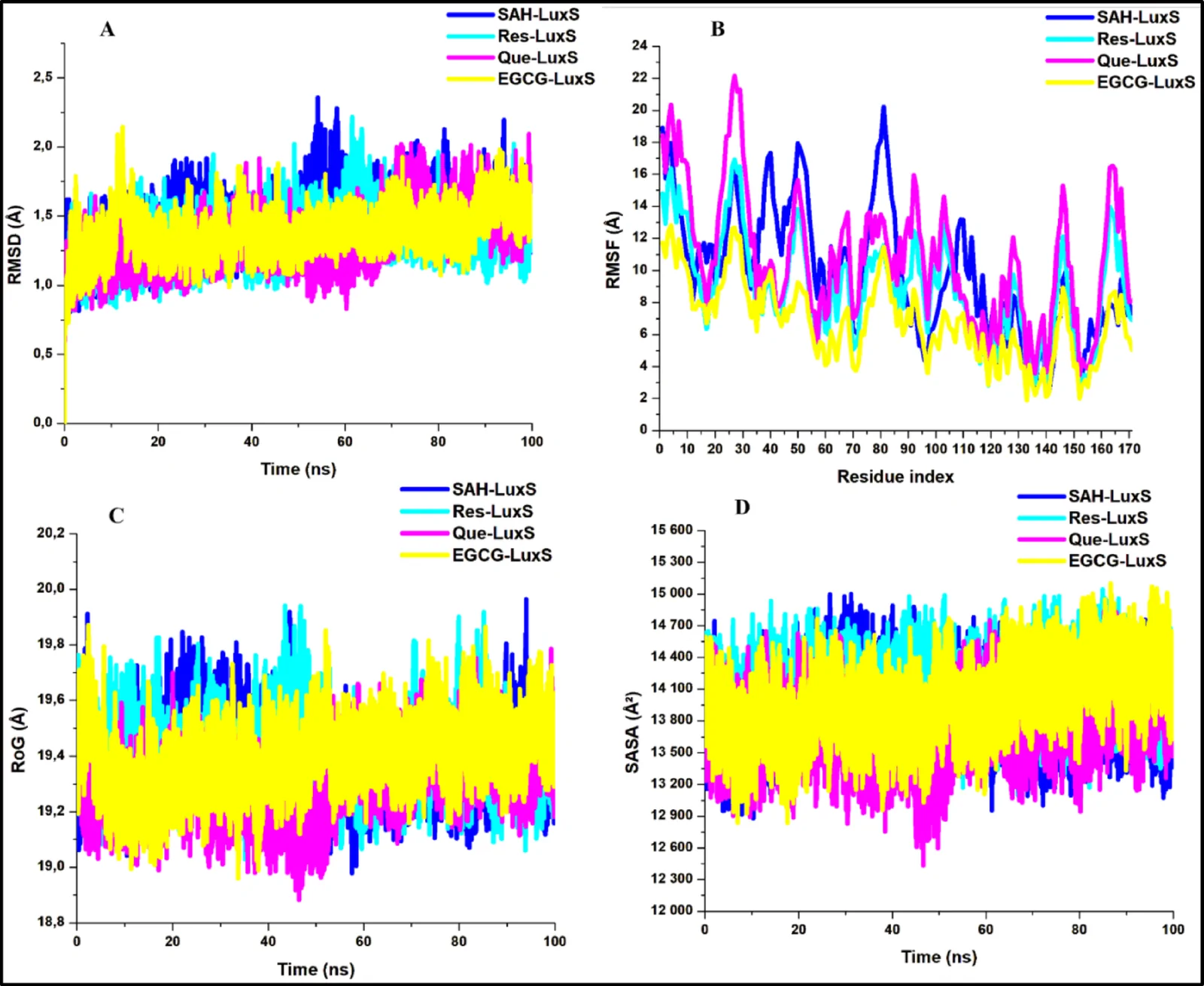
Structural dynamics of LuxS-ligand complexes. **A**: RMSD in Å calculated for Carbon backbone atoms in LuxS protein in complex with S-adenosylhomocysteine (SAH), Resveratrol (Res), Quercetin (Que) and Epigallocatechin gallate (EGCG); **B**: RMSF values in Å calculated for LuxS protein in complex with S-adenosylhomocysteine (SAH), Resveratrol (Res), Quercetin (Que) and Epigallocatechin gallate (EGCG); **C**: Rg values in Å calculated for LuxS protein in complex with S-adenosylhomocysteine (SAH), Resveratrol (Res), Quercetin (Que) and Epigallocatechin gallate (EGCG); **D**: SASA values in Å2 calculated for LuxS protein in complex with S-adenosylhomocysteine (SAH), Resveratrol (Res), Quercetin (Que) and Epigallocatechin gallate (EGCG) after 100 ns of MD simulations

Structural stability was assessed using the RMSD of Cα backbone atoms over the 100 ns trajectory (Fig. 3A). All complexes reached equilibrium within the initial phase of the simulation and remained stable throughout the trajectory. The LuxS-resveratrol complex exhibited the lowest deviation (1.8 ± 0.2 Å), indicating higher structural stability compared to other complexes. The LuxS-quercetin and LuxS-EGCG complexes showed slightly higher deviations (2.1 ± 0.3 Å and 2.4 ± 0.4 Å), suggesting comparatively flexible interactions. The SAH complex remained stable but displayed less compact behaviour over time. Residue-level flexibility analysis (Fig. 3B) revealed reduced fluctuations in key active-site regions (residues 50–70 and 120–140) for the LuxS-resveratrol complex, indicating stable binding interactions. In contrast, LuxS-EGCG exhibited the highest fluctuations in loop regions, suggesting less stable interaction over time. While the LuxS-SAH complex remained steady, LuxS-quercetin demonstrated intermediate flexibility, particularly in the peripheral loops.

The radius of gyration (Rg) analysis showed the LuxS–resveratrol complex maintained the most compact structure throughout the simulation, with an Rg value of 18.3 ± 0.1 Å compared with 19.4 ± 0.3 Å for the reference complex (Fig. 3C). Similarly, SASA analysis showed reduced solvent exposure for the LuxS–resveratrol complex with values above 15,500 Å² compared with the reference (Fig. 3D). These findings suggest that resveratrol promotes a more stable and compact protein conformation.

#### Hydrogen bond and principal component analysis (PCA)

**Fig. 4:**
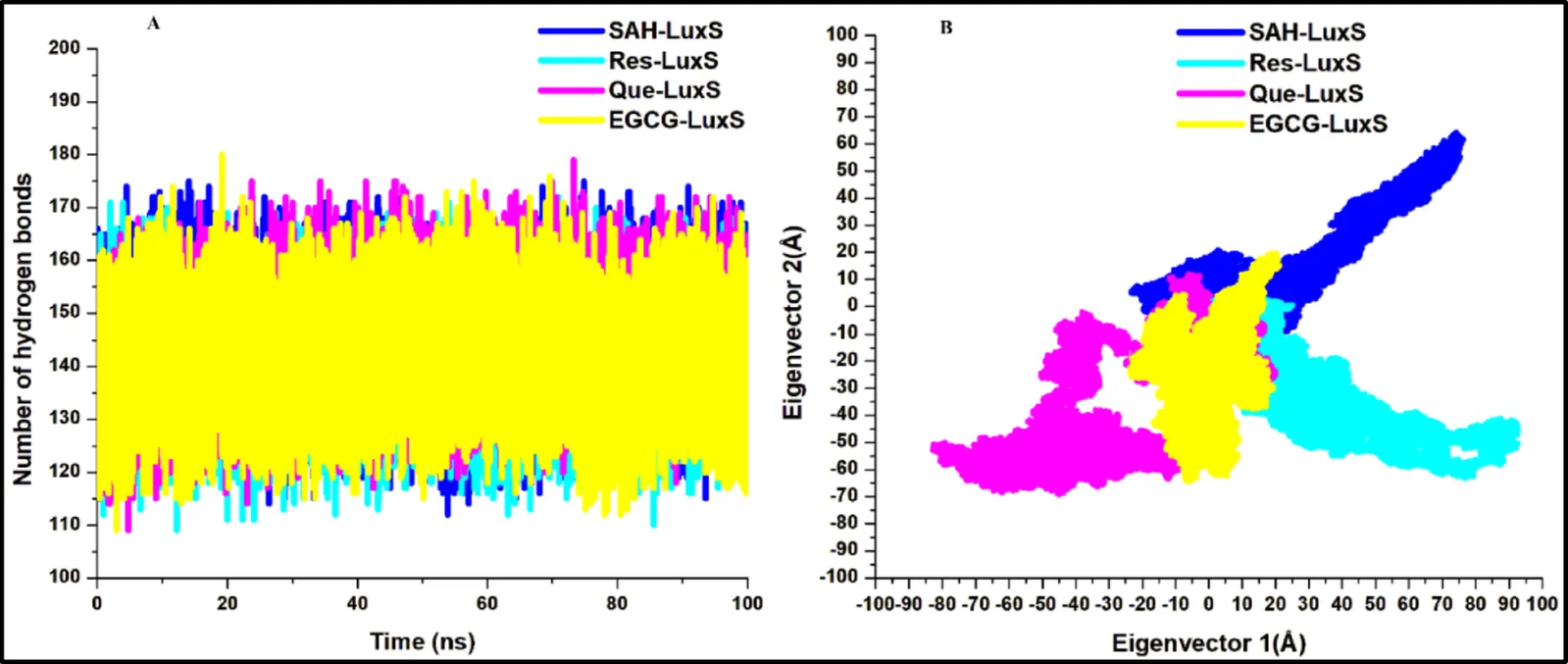
**A** 2-dimensional representation of intra-molecular hydrogen bond formation for LuxS protein in complex with S-adenosylhomocysteine (SAH), Resveratrol (Res), Quercetin (Que) and Epigallocatechin gallate (EGCG) calculated after 100 ns of MD simulations. **B** PCA plot constructed by eigenvector 1 vs. eigenvector 2 for for LuxS protein in complex with S-adenosylhomocysteine (SAH), Resveratrol (Res), Quercetin (Que) and Epigallocatechin gallate (EGCG) showing the positive and negative residual movements over 100 ns MD trajectories

Hydrogen bonding patterns were analysed over the final 100 ns of the trajectories (Fig. 4A). The LuxS–resveratrol complex formed stable hydrogen bonds with key active-site residues (e.g., Tyr63 and Trp68), along with π–π interactions that contributed to its stability. LuxS–quercetin showed fewer persistent interactions, while LuxS–EGCG exhibited more variable bonding patterns. The LuxS–SAH complex formed the fewest stable hydrogen bonds, correlating with reduced stability. Principal component analysis (PCA) was performed to characterise large-scale conformational motions. Projection onto PC1 and PC2 (Fig. 4B) revealed that the LuxS–resveratrol complex occupied a more confined conformational space, indicating restricted motion and enhanced stability. This restricted motion is indicative of enhanced stability and reduced structural flexibility. LuxS–quercetin and LuxS–EGCG showed broader distributions, reflecting greater conformational flexibility.

#### Secondary structure analysis

**Fig. 5:**
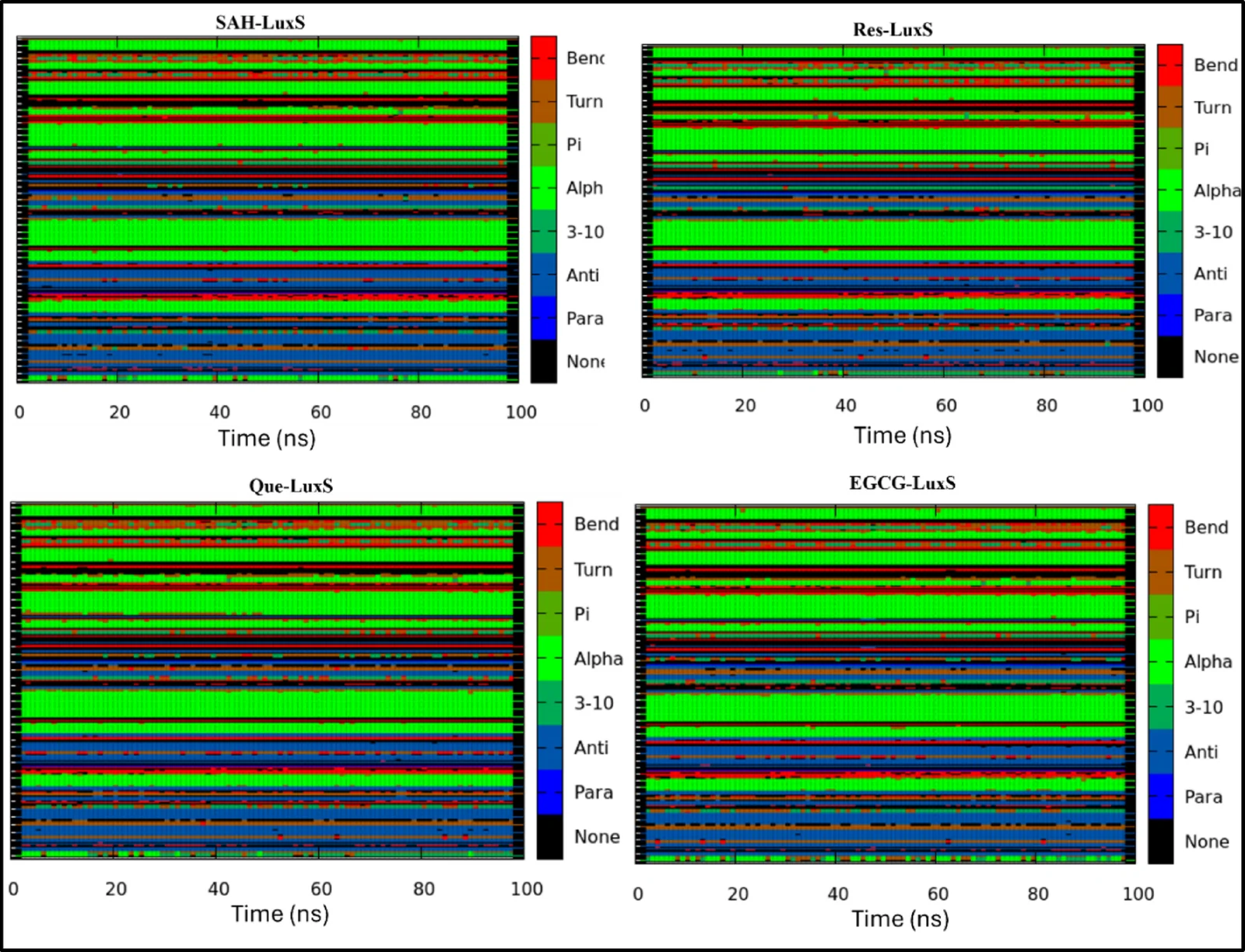
Secondary structure analysis of LuxS protein in complex with the studied compounds and the control ligand calculated after 100 ns of MD trajectories

Secondary structure analysis was performed to assess the impact of ligand binding on the structural integrity of the LuxS protein during the 100 ns simulation. Overall, all complexes retained their characteristic secondary structural elements, with α-helical and β-sheet regions remaining largely preserved throughout the trajectory. Secondary structure composition (Table 3) showed some variation among the complexes, with LuxS–quercetin exhibiting the highest α-helical content (26%), followed by LuxS–SAH and LuxS–EGCG (25%), while LuxS–resveratrol showed 23% α-helical content. These results indicate that ligand binding did not cause major disruption of the overall secondary structure of LuxS (Fig. 5).

The observed secondary-structure profiles, together with the RMSD and RMSF analyses, support the overall structural stability of the protein–ligand complexes.

**Table 3:** Percentage of residues contributing to the secondary structure of LuxS protein in complex with studied potential compounds and control ligand calculated after 100 ns of MD trajectories

Protein-ligand complex	α	Β	3_10_-Helix	Turn	Bend	Other
SAH-LuxS	25	19	12	12	13	19
Res-LuxS	23	24	8	15	14	16
Que-LuxS	26	26	11	9	13	15
EGCG-LuxS	25	23	10	11	12	19

#### Binding free energy calculations (MM/GBSA)

Binding free energies calculations showed that quercetin had the most favorable binding affinity (ΔG_bind = − 45.47 kcal/mol), followed by EGCG (–43.04 kcal/mol) and resveratrol (–39.22 kcal/mol), all outperforming the reference SAH (–33.04 kcal/mol). The strong binding affinity of quercetin and EGCG was primarily driven by favourable gas-phase interactions, including van der Waals and electrostatic contributions (Table 4), although these interactions were partially offset by solvation penalties. In contrast, resveratrol displayed a more balanced energetic profile, with strong van der Waals interactions and comparatively lower solvation penalties. Despite its slightly lower binding affinity, resveratrol demonstrated enhanced structural stability in MD simulation, highlighting the importance of considering both binding energetics and dynamic behaviour in ligand prioritisation. This observation suggests that dynamic stability may play a more important role than static binding affinity in determining biological activity. Although quercetin exhibited the most favourable MM/GBSA binding free energy, it did not demonstrate the strongest biological activity during experimental validation. This discrepancy indicates that binding free energy alone may not predict in vitro efficacy. Molecular dynamics analyses revealed that the LuxS–resveratrol complex maintained lower RMSD and RMSF values, reduced solvent exposure and greater structural compactness throughout the 100 ns simulation, indicating a more stable protein–ligand complex. Furthermore, resveratrol formed more persistent interactions with key active-site residues, suggesting enhanced conformational stability despite its slightly less favourable calculated binding free energy. In addition, the ADMET analysis predicted superior pharmacokinetic properties for resveratrol, including higher intestinal permeability and oral absorption, which may facilitate greater biological activity. Collectively, these findings demonstrate that integrating binding affinity with dynamic stability and predicted pharmacokinetic properties provides a more comprehensive framework for prioritising candidate compounds than reliance on docking or MM/GBSA scores alone.

**Table 4:** Binding free energy calculations of LuxS protein in complex with S-adenosyl-L-homocysteine (SAH), Resveratrol (Res), Quercetin (Que) and Epigallocatechin gallate (EGCG) using MM/GBSA approach

Protein-ligand complex	ΔEvdW	ΔEele	ΔGgas	ΔGpolar	ΔGnonpolar	ΔGsol	ΔGbind
SAH-LuxS	-45.39	-4.40	-49.79	21.12	-4.37	16.75	-33.04
Res-LuxS	-50.45	-5.87	-56.32	24.71	-7.61	17.10	-39.22
Que-LuxS	-57.31	-5.16	-62.47	23.71	-6.71	17.00	-45.47
EGCG-LuxS	-50.75	-8.30	-59.05	22.38	-6.37	16.01	-43.04

### In vitro validation

Based on the computational analysis, resveratrol, quercetin, and EGCG were selected for experimental validation. SAH was included as a reference compound to assess assay performance.

#### Antibacterial activity (viable cell reduction and MIC/MBC)

**Fig. 6:**
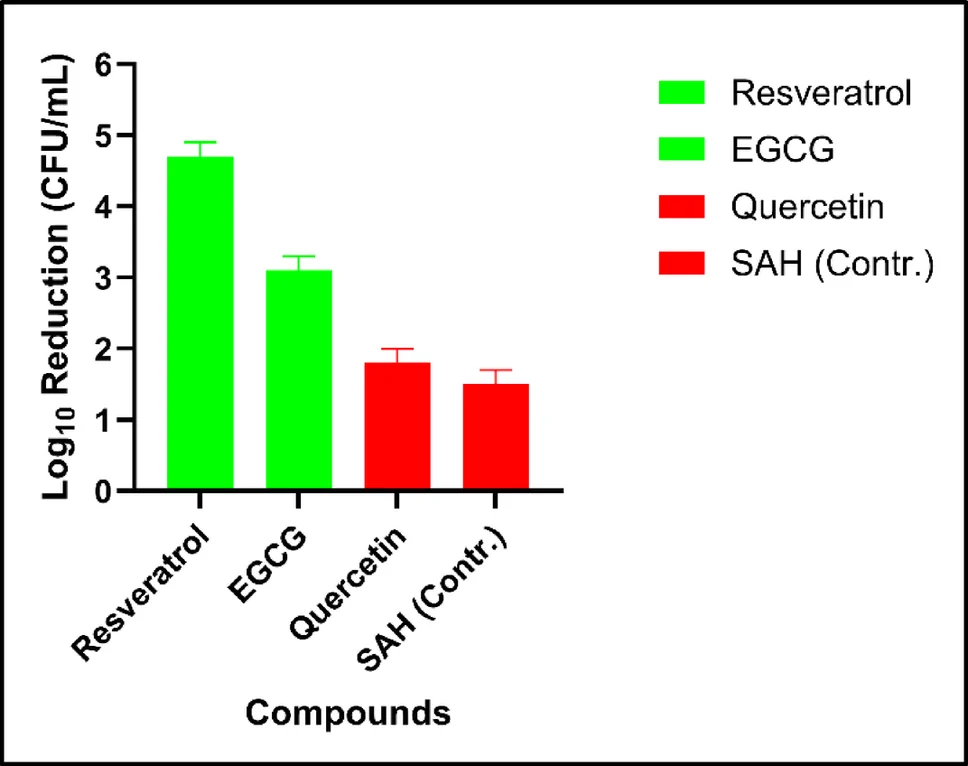
Effect of selected compounds on bacterial viability against *KPn* ATCC 13883: Bar chart showing log₁₀ reduction in viable CFU/mL at the Minimum Inhibitory Concentration (MIC). Compounds achieving ≥ 3 log₁₀ reduction (green bars) are classified as bactericidal, while those with < 3 log₁₀ reduction (red bars) are classified as bacteriostatic. The horizontal dashed line marks the bactericidal threshold (3 log₁₀ reduction). Resveratrol and EGCG demonstrated bactericidal activity, while quercetin and SAH were bacteriostatic. Data are presented as mean ± SD from three biological replicates (*n* = 3)

The antibacterial activity of the selected compounds was assessed using MIC and MBC assays and the results are summarised in Table 5; Fig. 6. Resveratrol exhibited the highest activity, achieving approximately a 5-log reduction in CFU/mL, indicating strong bactericidal effects. EGCG demonstrated moderate activity, with a reduction slightly above 3 logs, suggesting effective but comparatively lower antibacterial potency. In contrast, quercetin and SAH showed limited activity, with reductions below 2 logs. Consistent with these findings, MIC and MBC assays revealed that resveratrol exhibited the strongest antibacterial activity (MIC/MBC = 32 µg/mL), indicating bactericidal behaviour. EGCG also showed bactericidal activity, although at a higher concentration (256 µg/mL). In contrast, quercetin and SAH demonstrated bacteriostatic effects, with MIC values of 512 µg/mL and MBC values exceeding 1024 µg/mL (MBC: MIC > 2). Collectively, these results indicate that resveratrol is the most effective compound, demonstrating the highest antibacterial potency and reduction in bacterial viability among the tested compounds.

**Table 5:** Minimum Inhibitory Concentration (MIC) and Minimum Bactericidal Concentration (MBC) of Test Compounds Against *KPn* ATCC 13883 strain

Compound	MIC (µg/mL)	Log₁₀ Reduction at MIC	MBC (µg/mL)	MBC: MIC Ratio	Classification
Resveratrol	32	> 4.7	32	1	Bactericidal
EGCG	256	> 3.0	256	1	Bactericidal
Quercetin	512	1.7	> 1024	> 2	Bacteriostatic
SAH	512	1.7	> 1024	> 2	Bacteriostatic

#### Anti-quorum sensing activity

**Fig. 7:**
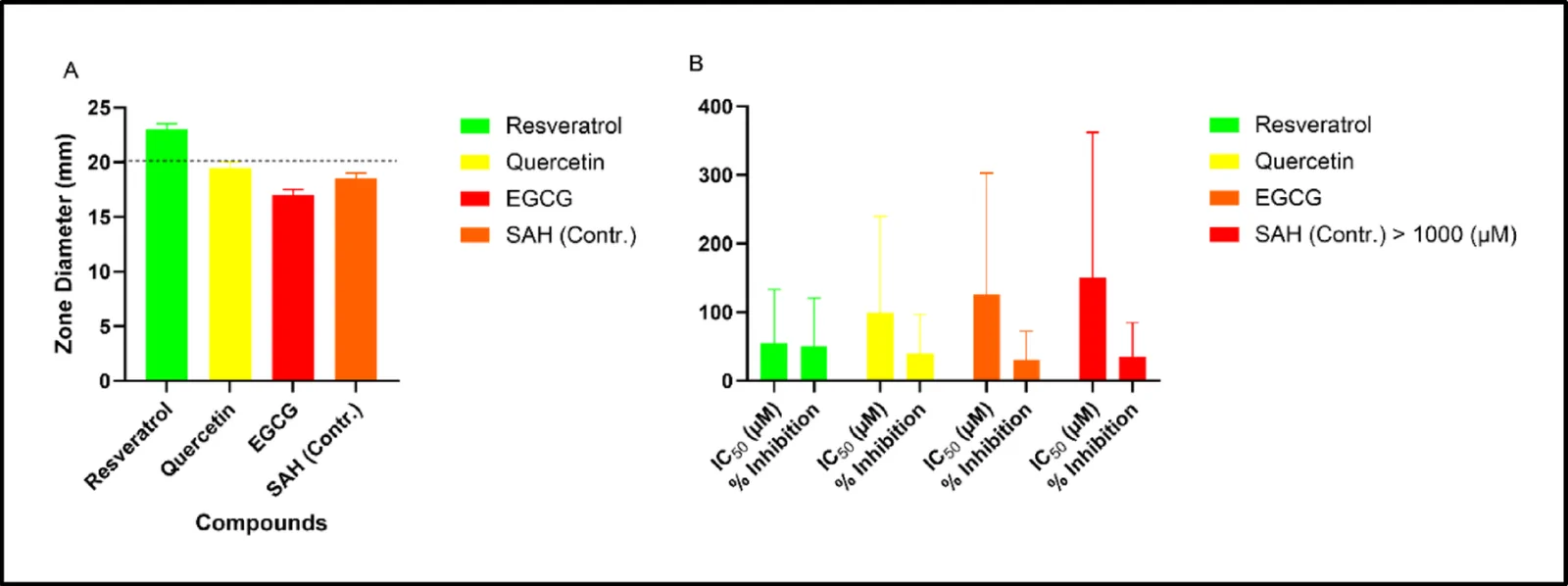
Quorum-sensing-associated phenotypic activity of selected compounds against *C. violaceum*. **A** Zones of violacein inhibition produced by the test compounds at MIC concentrations. **B** IC₅₀ values (µM) and percent violacein inhibition at MIC for each compound. Resveratrol showed the lowest IC₅₀ (110 µM) among the tested compounds. Data are presented as mean ± SD from three biological replicates (*n* = 3)

The anti-quorum sensing activity of the selected compounds was evaluated using the *C. violaceum* biosensor assay, in which inhibition of violacein production serves as a phenotypic indicator of quorum-sensing interference. All compounds produced measurable reductions in violacein production. Resveratrol exhibited the greatest activity, producing the largest zone of pigment inhibition (23.0 mm), with 99.8% inhibition at the tested concentration and the lowest IC₅₀ value (110 µM). Quercetin (19.5 mm) and EGCG (17.0 mm) demonstrated moderate activity, with IC₅₀ values of 199 µM (80% inhibition) and 251 µM (60% inhibition), respectively, whereas SAH showed comparatively weaker activity (Table 6; Fig. 7). However, because the initial violacein assays were performed at MIC concentrations, the observed reduction in violacein production may have been influenced by reduced bacterial growth or viability, particularly for resveratrol and EGCG, which exhibited bactericidal activity at their respective MICs (Table 5). Therefore, the present findings should be interpreted as evidence of quorum-sensing-associated phenotypic interference rather than definitive, growth-independent quorum-sensing inhibition. Although the observed activity is consistent with computational predictions and supports the prioritisation of resveratrol as a promising candidate, the *C. violaceum* biosensor primarily reflects AHL-mediated signalling and does not directly assess the LuxS/AI-2 pathway in *KPn*. Thus, these results should be interpreted as evidence of general quorum-sensing interference rather than direct evidence of the LuxS/AI-2 pathway inhibition in *KPn*. Future studies using non-growth-inhibitory sub-MIC concentrations, together with growth/viability measurements and biomass-normalised violacein production, will be required to confirm specific QS interference.

**Table 6:** Anti-quorum sensing activity of test compounds against *C. violaceum*

Compound	Zone Diameter (mm)	IC₅₀ (µM)	% Violacein Inhibition at MIC	Phenotypic QS Activity
Resveratrol	23.0	110	99.8%	Moderate
EGCG	17.0	251	60%	Weak
Quercetin	19.5	199	80%	Moderate (borderline)
SAH	18.5	> 1000	70% at 1000 μM	Very Weak (Reference)

#### Biofilm formation inhibition

**Fig. 8:**
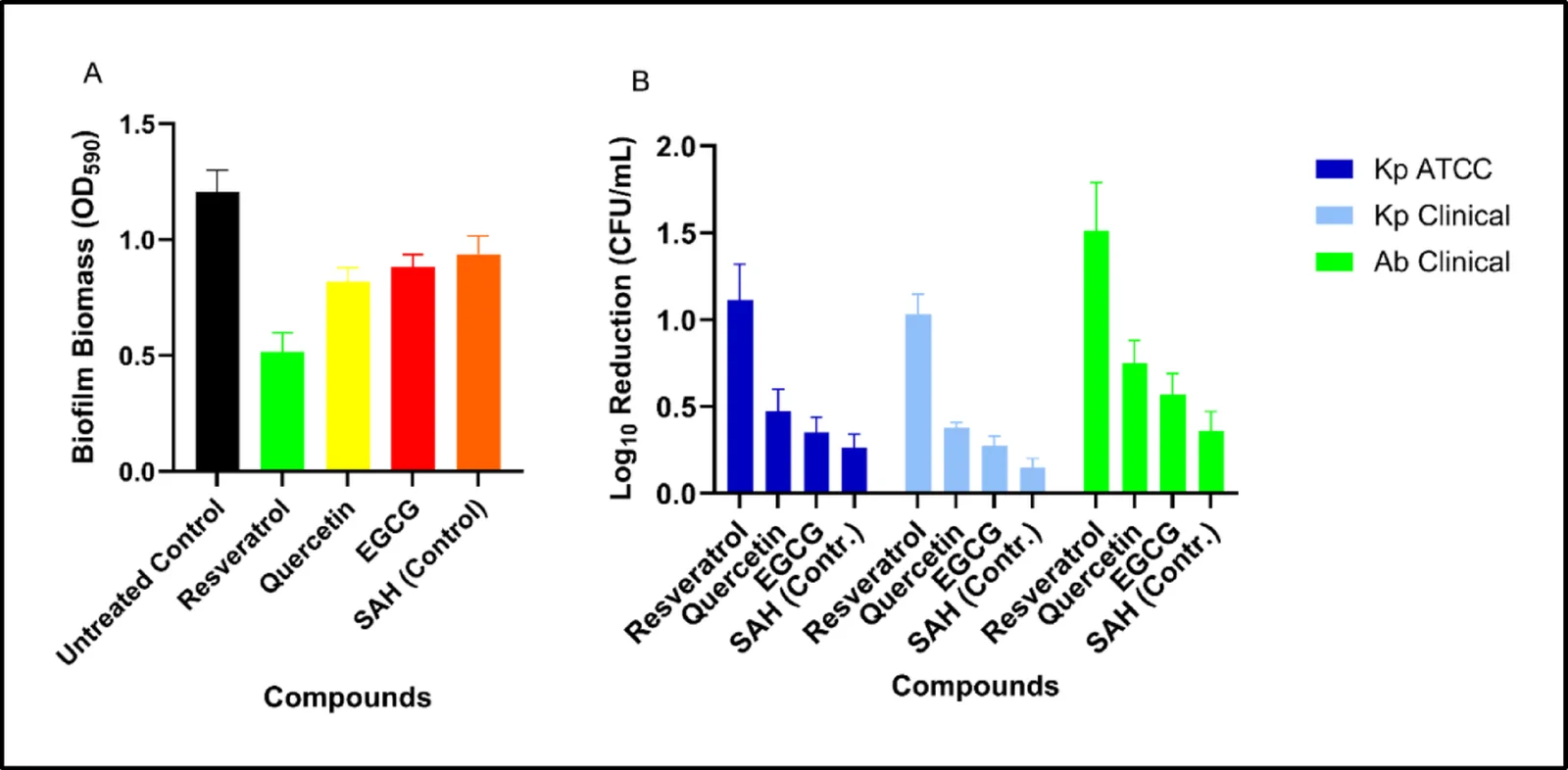
Effect of natural compounds on dual-species biofilm formation inhibition by *KPn* and *ABa*. **A** Biofilm biomass (OD₅₉₀) after treatment with natural compounds at 1/4 MIC. Resveratrol showed the highest inhibition (54.7%, green bar), followed, quercetin (35.2%), EGCG (29.7%), and SAH (20.3%). The horizontal dashed line indicates the 50% inhibition threshold for MBIC definition. **B** Log₁₀ reduction in viable counts of each species recovered on selective media. *ABa* (green bars) showed greater susceptibility to all compounds compared to *KPn* ATCC 13,883 (blue bars) and clinical isolate (light blue bars). Data are presented as mean ± SD from three biological replicates (*n* = 3). *p < 0.05, **p < 0.01, ***p < 0.001 compared to untreated control (one-way ANOVA with Dunnett’s post-hoc test)

Biofilm formation inhibition assays revealed that resveratrol significantly reduced dual-species biofilm formation at sub-MIC (8 µg/mL) concentrations, achieving over 50% inhibition. Quercetin (128 µg/mL) and EGCG (64 µg/mL) showed moderate effects with biomass inhibition of 35.2% and 29.7% respectively, while SAH exhibited minimal activity (1 mM; 20.3% inhibition) (Table 7; Fig. 8). Resveratrol also produced the greatest reduction in viable counts across the tested organisms. Interestingly, *ABa* showed greater reductions in viable counts than *KPn* following compound treatment, which may reflect differences in species-specific susceptibility, compound uptake, or biofilm-associated characteristics. However, the observed reduction in biofilm biomass may partly reflect the accompanying decrease in bacterial viability. Therefore, these findings should be interpreted as evidence of biofilm-associated phenotypic effects rather than definitive, growth-independent anti-biofilm activity. Future studies incorporating parallel planktonic growth controls and normalization of biofilm biomass to bacterial load will be required to distinguish specific anti-biofilm effects from reductions resulting from decreased bacterial viability.

**Table 7:** Effect of test compounds on dual-species biofilm formation inhibition and viable-cell reduction at 1/4 MIC

Compound	Concentration (1/4 MIC)	Biofilm Biomass (OD₅₉₀)	% Inhibition	Log₁₀ Reduction (KPn ATCC)	Log₁₀ Reduction (KPn Clinical)	Log₁₀ Reduction (ABa)
Untreated Control	-	1.28 ± 0.14	-	-	-	-
Resveratrol	8 µg/mL	0.58 ± 0.06	54.7%	1.1 ± 0.2	1.0 ± 0.2	1.6 ± 0.3
EGCG	64 µg/mL	0.90 ± 0.11	29.7%	0.4 ± 0.1	0.3 ± 0.1	0.6 ± 0.2
Quercetin	128 µg/mL	0.83 ± 0.10	35.2%	0.5 ± 0.2	0.4 ± 0.2	0.8 ± 0.2
SAH (Control)	1 mM	1.02 ± 0.15	20.3%	0.3 ± 0.1	0.2 ± 0.1	0.4 ± 0.2

## Conclusion

In this study, an integrative computational and experimental approach was employed to identify natural compounds with anti-quorum sensing and anti-biofilm activity against *Klebsiella pneumoniae*. Molecular docking, pharmacophore modelling, and molecular dynamics simulations prioritised resveratrol, quercetin, and epigallocatechin gallate as potential LuxS-targeting compounds. Although quercetin and epigallocatechin gallate exhibited stronger predicted binding affinities, resveratrol demonstrated enhanced structural stability and sustained interactions with key active-site residues. This stability was reflected in experimental validation, where resveratrol showed the strongest antibacterial activity, significant inhibition of quorum sensing-associated phenotypes, and substantial reduction in biofilm formation inhibition. These findings highlight the importance of integrating binding affinity with dynamic stability and biological validation when prioritising candidate compounds. The results support the potential of resveratrol as an anti-virulence agent that may interfere with quorum sensing and inhibit biofilm formation in multidrug-resistant pathogens. However, the present study does not provide direct experimental confirmation of LuxS inhibition. Overall, this study underscores the value of targeting quorum sensing as an alternative strategy to address antimicrobial resistance and highlights integrative computational-experimental approaches as effective tools for identifying novel anti-virulence therapeutics. Future investigations should include AI-2 quantification, LuxS enzyme inhibition assays and *luxS* gene expression analyses to confirm the proposed mechanism of action.

## Data Availability

The data used/generated to support the findings of this study are available from the corresponding author on reasonable request.
